# Appendiceal Intussusception Mimicking Cecal Mass and Fecal Matter: A Report of Two Rare Cases

**DOI:** 10.1155/2018/4809650

**Published:** 2018-07-16

**Authors:** James Haug, AS Katkar, James Covelli

**Affiliations:** San Antonio Military Medical Center, Ft. Sam Houston TX, USA

## Abstract

Intussusception of the appendix is a relatively rare event that is usually asymptomatic but can present similar to other acute and chronic abdominal conditions. We present two separate cases of suspected appendiceal intussusception mimicking cecal masses. The authors also present a cursory review of the limited number of literatures available concerning this entity to help the radiologist avoid misdiagnosis and potentially unnecessary invasive intervention.

## 1. Introduction

Abdominal pain is a very common, yet, nonspecific symptom. Abdominal pain is the most common symptom requiring a general surgery consultation. Specifically, appendicitis remains the most common surgical disease manifesting with abdominal pain and requiring emergent surgical intervention [1]. Presentation of intussusception of the appendix ranges from asymptomatic to an acute appendicitis-type picture. Patients may also complain of chronic abdominal pain, palpable abdominal mass, rectal bleeding, or constipation. Often laboratory data cannot distinguish appendiceal intussusception from other causes of abdominal pain [2].

There are different types of appendiceal intussusception (Types 1–5), according to the McSwain classification, which is anatomical, based on the region of the appendix that undergoes intussusception [3]. Type 1 involves invagination of the appendiceal tip. Type 2 is a more pronounced version of Type 1 with increased invagination of the appendiceal tip. Type 3 is the most common type and involves invagination at the junction of the appendix and cecum. Type 4 involves retrograde intussusception, where the proximal appendix is invaginated into the distal appendix. Type 5 is complete invagination of the appendix into the cecum.

Although no clear consensus on age of occurrence has been established, some studies have suggested that most events of appendiceal intussusception occur in the first decade of life. However, a literature review conducted on over 166 case reports concluded that this may in fact be a bimodal occurrence affecting middle age patients, in particular females [4].

Both normal and pathologic causes can result in appendiceal intussusception. Benign causes, such as a relatively more dilated proximal appendiceal lumen relative to the distal portion, mobile appendicular wall resulting in active peristalsis, or invagination of the appendiceal stump following appendectomy, are leading causes of appendiceal intussusception [5]. Suspicion for malignancy should be raised, in the absence of other important imaging findings or supporting clinical data. Malignancy of the appendix is rare, making up approximately 0.5% of all gastrointestinal tumors. These entities are usually carcinoid tumors, mucinous cystadenocarcinomas, colonic adenocarcinomas, and adenocarcinoid tumors [6].

### 1.1. Case Report 1

**Figure 1:**
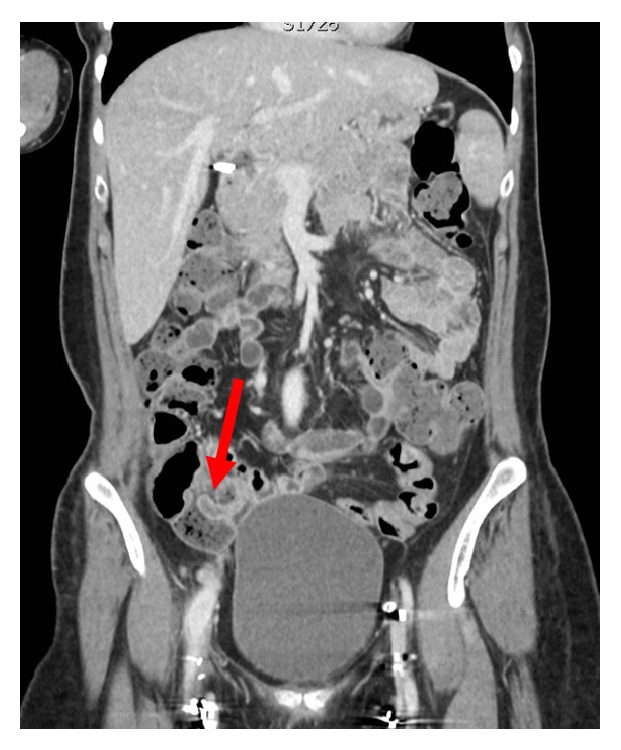
Coronal contrast enhanced CT image demonstrates a long, hypodense tubular structure representing a normal appendix (arrow) invaginating in the lumen of the cecal base.

**Figure 2:**
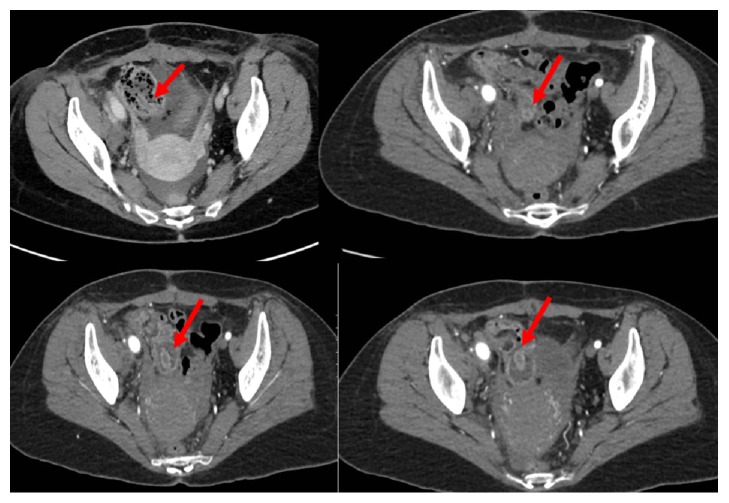
Multiple contrast enhanced axial images of the pelvis demonstrate a circular hypodense structure arising from the base of the cecum extending into the fluid-filled cecal lumen. Incidentally noted pelvic ascites are present.

We present a case involving a 40-year-old female who was found to have a McSwain Type 5 inverted appendix on a computed tomography (CT) urogram for hematuria and flank pain. A review of her past imaging included a computed tomography (CT) aorta and abdomen/pelvis which also revealed this anatomic variant and appeared unchanged over the span of approximately nine months (Figures 1 and 2). Laboratory values to include white blood cell, red blood cell count, and lactate levels revealed no significant abnormality. The only laboratory derangement involved elevated transaminases, which were felt to be unrelated to her acute presenting symptoms. The patient did not report any history of a prior appendectomy. Her only relevant surgical history involved thrombolysis of the right common iliac artery and subsequent stenting of the left internal iliac vein due to compressive symptoms associated with May-Thurner syndrome. The patient did not undergo surgery and her initial presenting symptoms were felt to be unrelated to this imaging finding.

### 1.2. Case Report 2

A 35-year-old male presented to the emergency department with nonspecific abdominal pain. The patient was afebrile with normal laboratory values to include normal lactate. The patient did not have any history of malignancy or other chronic medical conditions. Contrast enhanced computed tomography was performed, which demonstrated a fluid-filled polypoid mass within the base of the cecum (Figure 4). No other concerning imaging findings were reported. A colonoscopy was performed which revealed a bulge in the cecum. No abnormal mass or inflammatory signs were observed. Findings were favored to represent an invaginated appendix, or cecoappendiceal intussusception, rather than a colonic mass and a biopsy was deferred. The patient was treated with bowel rest and antibiotics. She was discharged from the hospital after a few days with followup scheduled with gastroenterology.

## 2. Discussion

Inverted appendix is a rare occurrence that is poorly understood amongst clinicians and diagnostic radiologists. As a result, it is often overlooked or mistaken for other pathologic processes in patients presenting with nonspecific abdominal pain without any other obvious pathology. To make matters more difficult, not all cases of appendix intussusception are symptomatic. However, when symptomatic, the presentation is most frequently nonspecific and chronic in nature.

Inverted appendix diagnosed on colonoscopy has typically been associated with benign conditions such as endometriosis or chronic appendicitis. However, the surgical literature suggests presence of an associated underlying malignancy on postoperative pathologic review in 43% of patients with an inverted appendix [6].

Currently there are no guidelines to suggest if further evaluation for this entity is warranted. The approach can range from simple appendectomy to right colectomy if there are signs of obstruction. Intussusception tends not to respond permanently to nonsurgical treatment, and simple appendectomy may not be adequate treatment. Other alternative surgical procedures include appendiceal inversion, which can mimic an inverted normal appendix and be misdiagnosed if pertinent surgical history is not elicited [7, 8].

**Figure 3:**
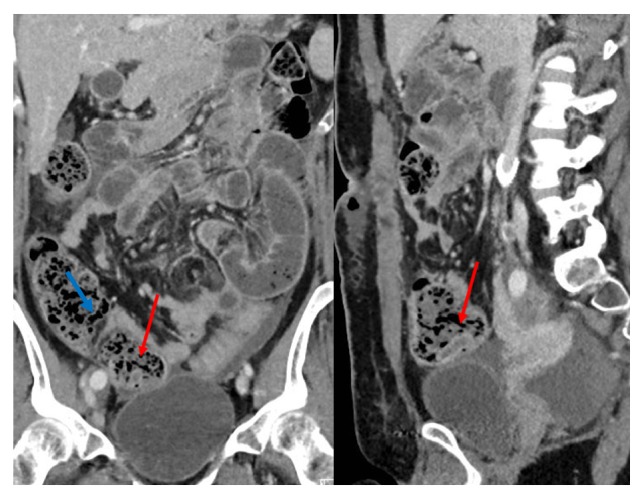
Contrast enhanced coronal and sagittal computed tomography (CT) image of the abdomen and pelvis of Patient 1 at another time reveals unchanged normal appendix (red arrow) invaginated into the cecum, now surrounded by fecal particulate matter, making detection more difficult. Note the submucosal fat from the ileocecal valve (blue arrow).

**Figure 4:**
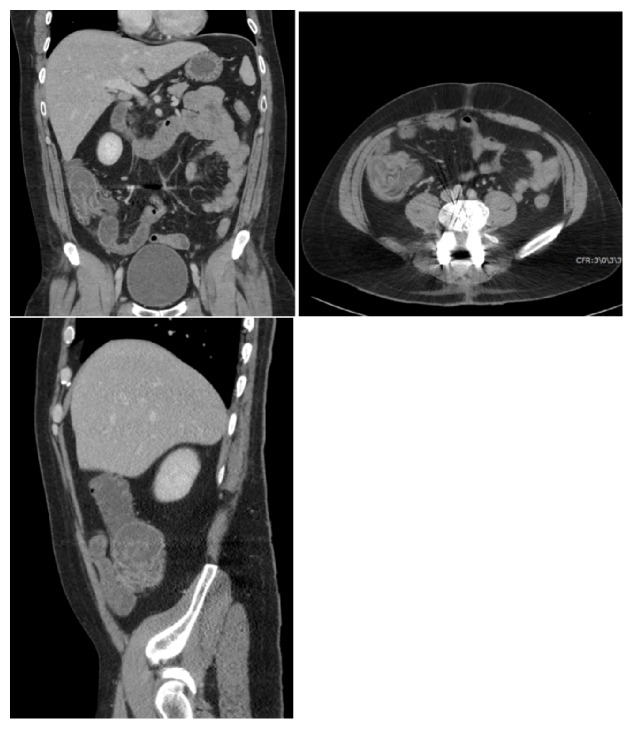
Coronal, axial, and sagittal contrast enhanced CT image demonstrates a polypoid mass in the base of the cecum representing a cecoappendiceal intussusception, mimicking a polyp or neoplasm.

Imaging plays a large, important role as the number of variations and nonspecific symptoms can make it difficult to accurately detect the presence or absence of appendiceal pathology. This is further complicated if fecal particulate matter surrounds the invaginated appendix, masking the appendix, as was the case for our first patient. However, on CT, a target, layered, sausage-shaped, or reniform appearance, when present, is virtually pathognomonic. Difficulty in detecting an inverted appendix can be exacerbated if the adjacent cecum is filled with feces as we saw in our patient (Figure 3). An appendiceal inversion may appear on endoscopy to be a polypoid area covered with normal mucosa, giving the appearance of an adenomatous polyp, with a central dimple found in the anatomic place of the appendix. Sonographic findings include multiple concentric hyperechoic and hypoechoic rings. On barium studies, a characteristic coiled-spring appearance in the cecum with nonfilling of the appendix has been described in 11 cases of apparent or proved appendiceal intussusception [9].

Appendiceal intussusceptions are pathologically different and prognostically distinct. As such, prompt and accurate recognition of this entity by the radiologist is imperative to avoid an unnecessary surgery that could potentially result in an endoscopic biopsy, a relatively invasive procedure that could lead to perforation and subsequent peritonitis.

## 3. Conclusion

Intracecal positioning of the normal appendix is a rare occurrence and can be symptomatic. Awareness of anatomic variations and mimics can assist the radiologist in making an accurate diagnosis and avoiding relatively invasive interventions and unnecessary surgery.
